# The cost of migration: spoonbills suffer higher mortality during trans-Saharan spring migrations only

**DOI:** 10.1098/rsbl.2014.0944

**Published:** 2015-01

**Authors:** Tamar Lok, Otto Overdijk, Theunis Piersma

**Affiliations:** 1 Chair in Global Flyway Ecology, Animal Ecology Group, Centre for Ecological and Evolutionary Studies, University of Groningen, PO Box 11103, 9700 CC Groningen, The Netherlands; 2 Department of Marine Ecology, NIOZ Royal Netherlands Institute for Sea Research, PO Box 59, 1790 AB Den Burg, Texel, The Netherlands; 3 Werkgroep Lepelaar, 9166 NZ Schiermonnikoog, The Netherlands

**Keywords:** seasonal survival, mark–recapture analysis, differential migration, evolution of migration, long-distance migration, Sahara desert

## Abstract

Explanations for the wide variety of seasonal migration patterns of animals all carry the assumption that migration is costly and that this cost increases with migration distance. Although in some studies, the relationships between migration distance and breeding success or annual survival are established, none has investigated whether mortality during the actual migration increases with migration distance. Here, we compared seasonal survival between Eurasian spoonbills (*Platalea leucorodia leucorodia*) that breed in The Netherlands and migrate different distances (*ca* 1000, 2000 and 4500 km) to winter in France, Iberia and Mauritania, respectively. On the basis of resightings of individually marked birds throughout the year between 2005 and 2012, we show that summer, autumn and winter survival were very high and independent of migration distance, whereas mortality during spring migration was much higher (18%) for the birds that wintered in Mauritania, compared with those flying only as far as France (5%) or Iberia (6%). As such, this study is the first to show empirical evidence for increased mortality during some long migrations, likely driven by the presence of a physical barrier (the Sahara desert) in combination with suboptimal fuelling and unfavourable weather conditions *en route*.

## 1. Introduction

Migration—the regular seasonal movement of individuals, often from a breeding location to a non-breeding location and back—is a common and taxonomically widespread phenomenon throughout the animal kingdom [1]. Migration is considered to have evolved as an adaptation to exploit seasonal peaks in resource abundance while avoiding seasonal resource depression during the non-breeding period by travelling—at some cost—to more benign areas. There is much variation in migration patterns, between and within species, and even within (breeding or wintering) populations of the same species [2–3]. As reviewed in [4], many theories have been developed to explain this enormous variation in migration patterns. These theories all assume that migration is costly and that this cost increases with migration distance. This cost may be direct, causing reduced survival during migration [5,6], or it may carry over to the next season, reducing subsequent survival or reproductive output.

While some studies have correlated individual migration distances with timing of spring arrival and breeding performance [7–10], none has investigated whether longer migrations were associated with higher direct or delayed mortality rates. This paucity of studies is easily explained by the difficulty of following individual birds throughout their annual cycle. Although some studies compared annual survival of individuals or populations with varying migration distances [11,12], estimating annual survival is not appropriate for measuring the cost of migration; the mortality cost of migration may be outweighed by the survival benefits of wintering further away from the breeding grounds [13]. For a proper assessment of the mortality cost of migration, we need to compare mortality during migratory and stationary periods of individuals with varying migration distances, while keeping constant as many other variables as possible.

**Figure 1.**
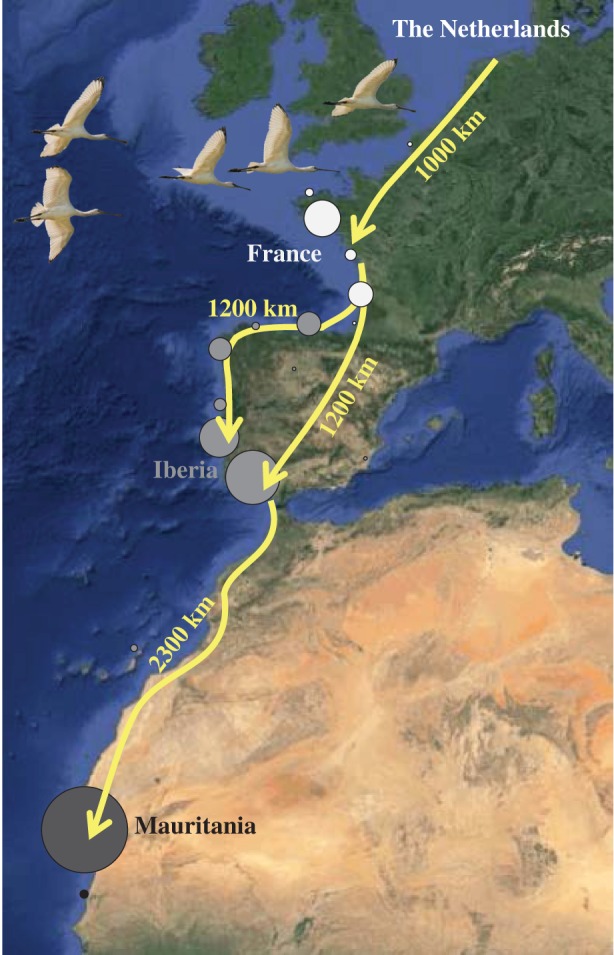
Map of spoonbill migration system. The sizes of the dots represent the number of individuals in the analysis that wintered at each site. (Online version in colour.)

Here, we make such a comparison of seasonal survival by comparing individuals that share their breeding area and flyway, yet show considerable variation in migration distances and wintering areas (hereafter called ‘migration strategies'). Our study system is the northwest European population of Eurasian spoonbills *Platalea leucorodia leucorodia* that breed in The Netherlands and winter along the East-Atlantic coast between France and Senegal (figure 1). Spoonbills are faithful to their wintering area [14] and this allows the comparison of seasonal survival rates among individuals with different migration strategies. We test whether (i) mortality during migration increases with migration distance (direct cost of migration), (ii) mortality during the breeding season increases with migration distance (delayed cost of migration) and (iii) mortality during winter decreases with migration distance (benefit of wintering in tropical areas).

## 2. Material and methods

This study uses summer and winter resightings between 2005 and 2012 of Eurasian spoonbills that were individually colour-banded as chicks in The Netherlands, for which we relied on a network of dedicated amateur and professional ornithologists, supplemented with spoonbill resighting expeditions to the Banc d'Arguin in Mauritania. We selected data on seasonally migrating adult birds (≥ third winter) that were observed at least once in their wintering area during the study period (between October and March in Mauritania or between December and January in Europe, to exclude resightings during stopover to more southern wintering sites), excluding the 27 birds that switched sites between winters. This resulted in 538 individuals, of which 65 wintered in France (one-way migration distance ≈ 1000 km), 236 in Iberia (migration distance ≈ 2000 km) and 237 in Mauritania (migration distance ≈ 4500 km) (figure 1).

To distinguish between mortality during summer, autumn migration, winter and spring migration, we used resightings at the start and end of summer (April–June and July–September) in the breeding areas in The Netherlands and Germany and at the start and end of winter (October–December and January–March) in an individual's wintering area. Because individuals were only included in the analysis when observed at least once in winter, their encounter histories started with their first winter observation. To separate apparent survival (*Φ*) from resighting probabilities (*p*), we used Cormack–Jolly–Seber models [15]. Based on previous findings [16] and to test our predictions, we built a set of candidate models and used QAIC_c_ (Akaike information criterion, adjusted for small sample size and overdispersion (median *ĉ* = 1.34) [17]) to assess their relative support. We reported parameter estimates and profile likelihood confidence intervals from the best-supported model. Further details of the methods are described in the electronic supplementary material.

## 3. Results

**Figure 2.**
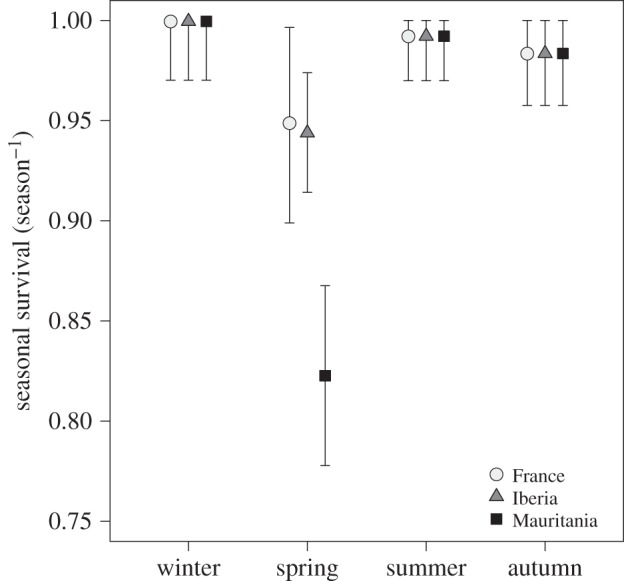
Seasonal survival of birds wintering in France, Iberia and Mauritania.

Survival differed between the seasons and was especially low during spring migration (table 1 and figure 2). There was also a strong effect of migration strategy on spring migration survival (the sum of Akaike weights of models including the effect (∑*w_i_*) = 1.00, table 1), with birds wintering in Mauritania being three times more likely to die during spring migration (17.7%) than birds wintering in France (5.1%) or Iberia (5.6%) (figure 2). This result was not explained by an incidental mass mortality of Mauritanian winterers during spring migration [18], as modelling annual variation revealed that the pattern was consistent in four of the six years. During the other three seasons, survival was very high and independent of migration strategy (∑*w_i_* = 0.41, 0.33 and 0.29 for the models including an effect of migration strategy on autumn, winter and summer survival, respectively; table 1 and figure 1).

**Table 1.** Model selection results. K = number of parameters; *Φ* = apparent survival; c = constant; m = migration strategy. Resighting probability is modelled the same in all models (see the electronic supplementary material). *Qdeviance = 4616.95. **QAIC_c_ = 6681.76.

model		K	ΔQdeviance	ΔQAIC_c_	Akaike weight
(1)	*Φ*^summer^_c_ *Φ*^autumn^_c_ *Φ*^winter^_c_ *Φ*^spring^_m_	50	8.06	0.00**	0.27
(2)	*Φ*^summer^_c_ *Φ*^autumn^_m_ *Φ*^winter^_c_ *Φ*^spring^_m_	52	4.33	0.42	0.21
(3)	*Φ*^summer^_m_ *Φ*^autumn^_c_ *Φ*^winter^_c_ *Φ*^spring^_m_	52	5.26	1.35	0.13
(4)	*Φ*^summer^_c_ *Φ*^autumn^_c_ *Φ*^winter^_m_ *Φ*^spring^_m_	52	5.53	1.62	0.12
(5)	*Φ*^summer^_c_ *Φ*^autumn^_m_ *Φ*^winter^_m_ *Φ*^spring^_m_	54	1.47	1.71	0.11
(6)	*Φ*^summer^_m_ *Φ*^autumn^_c_ *Φ*^winter^_m_ *Φ*^spring^_m_	54	2.49	2.74	0.07
(7)	*Φ*^summer^_m_ *Φ*^autumn^_m_ *Φ*^winter^_c_ *Φ*^spring^_m_	54	2.87	3.11	0.06
(8)	*Φ*^summer^_m_ *Φ*^autumn^_m_ *Φ*^winter^_m_ *Φ*^spring^_m_	56	0.00*	4.40	0.03
(9)	*Φ*^summer^_m_^=^^winter^ *Φ*^autumn^_m_^=^^spring^	50	23.56	15.51	0.00
(10)	*Φ*^summer^_m_^=^^autumn^^=^^winter^^=^^spring^	47	34.14	19.87	0.00
(11)	*Φ*^summer^_c_ *Φ*^autumn^_c_ *Φ*^winter^_m_ *Φ*^spring^_c_	50	29.34	21.28	0.00
(12)	*Φ*^summer^_m_ *Φ*^autumn^_c_ *Φ*^winter^_c_ *Φ*^spring^_c_	50	29.77	21.72	0.00
(13)	*Φ*^summer^_m_ *Φ*^autumn^_c_ *Φ*^winter^_m_ *Φ*^spring^_c_	52	27.08	23.17	0.00
(14)	*Φ*^summer^_c_ *Φ*^autumn^_m_ *Φ*^winter^_m_ *Φ*^spring^_c_	52	28.64	24.73	0.00
(15)	*Φ*^summer^_m_ *Φ*^autumn^_m_ *Φ*^winter^_c_ *Φ*^spring^_c_	52	29.72	25.81	0.00
(16)	*Φ*^summer^_m_ *Φ*^autumn^_m_ *Φ*^winter^_m_ *Φ*^spring^_c_	54	26.35	26.59	0.00
(17)	*Φ*^summer^_c_ *Φ*^autumn^_m_ *Φ*^winter^_c_ *Φ*^spring^_c_	50	34.65	26.59	0.00
(18)	*Φ*^summer^_c_ *Φ*^autumn^_c_ *Φ*^winter^_c_ *Φ*^spring^_c_	48	39.83	27.63	0.00
(19)	*Φ*^summer^_c_^=^^winter^ *Φ*^autumn^_c_^=^^spring^	46	52.86	36.53	0.00
(20)	*Φ*^summer^_c_^=^^winter^^=^^autumn^^=^^spring^	45	58.48	40.09	0.00

These findings are partially consistent with prediction (i) that mortality during migration increases with increasing migration distance. Predictions (ii) and (iii) are rejected, as summer survival of Mauritanian winterers was not lower (i.e. no evidence for a carry-over effect of long-distance migration on the probability of survival) and winter survival not higher (no survival benefit of wintering further south).

In absence of a survival benefit of wintering further south, the annual survival was lower for Mauritanian winterers (0.80, 0.75–0.85) compared with birds wintering in France (0.93, 0.87–0.96) or Iberia (0.92, 0.89–0.94). Resighting probability estimates are shown in electronic supplementary material, figure S1.

## 4. Discussion

The main result of this study is that mortality during spring migration was higher in the longest distance (trans-Sahara) migrating spoonbills that wintered in Mauritania, compared with those staying in Europe. This finding partially supports the general assumption of theories aimed to explain the evolution of migration, i.e. that the cost of migration increases with the distance migrated. However, it also shows that there is more to it than distance, as (i) mortality of trans-Saharan migrants was not higher during autumn migration, and (ii) birds wintering in Iberia did not have higher migration mortality compared with the birds wintering in France.

The fact that birds wintering in Mauritania had much higher mortality during spring migration than the European winterers suggests that the main spring migration mortality of Mauritanian winterers occurred during the crossing of the Sahara (an observation made in other migrants too [5,19]). When crossing the Sahara during northward migration, trade winds cause a high incidence of headwinds at lower altitudes of flight, up to *ca* 2 km height [20]. This would reduce ground speed and increase the necessary time in the air. Conversely, these trade winds result in a high incidence of tailwinds during autumn migration, which may explain the absence of increased mortality during autumn migration. While this scenario may seem specific to birds that cross the Sahara during migration, it is the result of two potentially general mechanisms that could drive migration–distance related patterns in mortality during migration, being that (i) longer migrations are more likely to include the crossing of physical barriers (e.g. deserts, mountain ranges or oceans) and (ii) birds migrating longer distances are more likely to encounter adverse weather conditions during migration, simply because they make more flight hours [18]. Yet, these factors are expected to reduce survival only if birds are unable to anticipate such challenges by depositing sufficiently large nutrient stores prior to departure.

The ability to deposit sufficient nutrient stores may depend on a species' morphology, physiology, on the ecological conditions at departure and staging sites and on the distances between suitable staging sites. For example, the generally higher mortality of spoonbills during spring than autumn migration, also for those wintering in Europe that are not affected by trade winds, could indicate that ecological conditions were less favourable for (re)fuelling during spring than during autumn. The few other studies on long-distance migrating species that were able to (partially) separate mortality during migratory and stationary periods also indicate that patterns of seasonal mortality may be species- or context-specific. Similar to the spoonbills, most mortality of a long-distance migrating passerine [6] and several species of raptors [5] occurred during migration. By contrast, most mortality of red knots *Calidris canutus canutus*, a shorebird species that is physiologically capable of storing the nutrients necessary for up to 9000 km non-stop flights, occurred on the non-breeding grounds [21]. Clearly, more studies on species with varying morphology, physiology, migration routines and ecological contexts are needed to quantify the physical, physiological and ecological drivers that shape patterns of migration-associated mortality.

## Supplementary Material

Supplementary_material

## Data accessibility

The data underlying this study are available on Dryad: doi:10.5061/dryad.kt845.
