# Supplementary material for: The cost of migration: spoonbills suffer higher mortality during trans-Saharan spring migrations only
Source: Biol Lett. 2015 Jan;11(1):20140944. doi: 10.1098/rsbl.2014.0944 (PMC4321157; doi:10.1098/rsbl.2014.0944)
Supplement: Supplementary_material [file rsbl20140944supp1.docx]

# Electronic Supplementary Material

## Methods

Eurasian spoonbills *Platalea leucorodia leucorodia* have been colour-ringed as pre-fledged chicks in The Netherlands since 1982 and resighted throughout the year and across their geographical range between Denmark in the north and Senegal in the south. For resightings in the European part of their geographical range, we relied on a large network of dedicated amateur and professional ornithologist. These resightings were sufficient to separate mortality during the winter season from mortality during the migratory seasons for spoonbills wintering in Europe. To be able to make this separation as well for birds wintering in Mauritania, additional expeditions were organized to the Banc d’Arguin (Mauritania) in early winter (Dec 2005, Oct-Nov 2006, 2007, 2008) and late winter (Jan 2006, 2008, 2009, 2011, 2012) with the specific aim to read spoonbill colour-rings. In addition, we benefited from expeditions to the Banc d’Arguin in December 2006-2011 by other researchers that performed ring-reading of spoonbills aside their main research activities (e.g., [1-2]).

As a result, we had sufficient resightings at the start and end of the winter season between 2005 and 2012 to compare seasonal survival for birds wintering in France, Iberia (Spain and Portugal) and Mauritania. Due to lack of seasonal resightings in Senegal, we excluded birds wintering there. We only used data on adult birds (≥ 3^rd^ winter) as these are assumed to perform seasonal migrations, in contrast to younger birds that stay at the wintering grounds year-round. Following Lok *et al.* [3], the wintering area of an individual was defined as the area where it was observed in the winter period, defined as December and January for France or Iberia (to minimize the probability that an individual was observed at a stopover rather than its wintering site) and October to March for Mauritania (not used as a stopover). Most spoonbills remain faithful to a single wintering area [3], yet 27 birds switched wintering sites between winters and were excluded from the analysis.

Seasonal survival was estimated using Cormack-Jolly-Seber models that enable the separation of apparent (or local) survival (Φ) and resighting probability (p) [4]. The defined resighting periods April-June (s1) and July-September (s2) at the breeding areas and October-December (w1) and January-March (w2) at an individual’s wintering area allowed us to estimate survival during four 3-month seasons: summer (half May – half August), autumn migration (half August – half November), winter (half November – half February) and spring migration (half February – half May). Although the resighting periods are as long as the intervals over which survival is being estimated, the bias incurred from this has been shown to be relatively small, especially when mortality and resighting probabilities are lower than 50% [5]; in fact, the pooling of resightings may even increase the precision of the survival estimates [6]. Moreover, the fact that we only used resightings at the breeding and wintering grounds ascertains that mortality during migration does not overlap with the period of resighting. The encounter history data are summarized in table S1.

Based on previous results [7], we included annual variation in resighting probability during the summer periods and during the winter periods in Iberia and Mauritania. Due to limited data, we modelled constant resightings probabilities for the winter periods in France. In addition, we included an additive effect of migration strategy on resighting probabilities during summer. Our starting model was ϕ^summer^_m_ ϕ^autumn^_m_ ϕ^winter^_m_ ϕ^spring^_m_ p^s1^_t+m_ p^s2^_t+m_ p^w1,F^_c_ p^w1,I^_t_ p^w1,A^_t_ p^w2,F^_c_ p^w2,I^_t_ p^w2,A^_t_, where the additive effect of migration strategy was the same for p^s1^ and p^s2^, for which we tested goodness-of-fit using the median-ĉ procedure implemented in program MARK [8]. The level of overdispersion was estimated at 1.34 ± 0.01.

Some ϕ parameters in the full model, but also in reduced models, were estimated at the boundary of 1. There may be several reasons for the occurrence of a boundary parameter: (i) it is not uniquely identifiable (intrinsic identifiability), (ii) it is not estimable due to lack of data (extrinsic identifiability), or (iii) this parameter is truly at the boundary. Given the structural simplicity of our models (single state and no year-to-year variation in seasonal survival parameters) all parameters were intrinsically identifiable. To test whether the boundary parameters were extrinsically identifiable, we applied data cloning (see the manual of program MARK for a description of the procedure [9]). Reported confidence intervals of all ϕ parameters were derived using the profile likelihood function.

Annual survival estimates were calculated from the seasonal estimates using the delta method based on the variance-covariance matrix on the probability (not logit) scale to derive the profile likelihood confidence intervals.

Models were constructed in R v. 2.13.0 [10] using package RMark [11] and run using program MARK [8].

## Results

All Φ parameters were estimable for the full model (table S1) as well as the best-supported model (table S2), indicated by the fact that all 95% profile likelihood confidence intervals were reduced when data was cloned 100 times.

Resighting probabilities in winter were generally higher at the start (w1) than at the end (w2), and were highest in France and lowest in Mauritania (figure S1). Summer resighting probabilities varied strongly between years, and this year-to-year variation differed between the start (s1) and end (s2) of the summer period*.* Moreover, similar to previous findings [7], summer resighting probabilities were higher for European than for Mauritanian winterers.

**Table S1.** Summary of encounter history data, showing the number of birds “released” (i.e. observed for the first time in their wintering area) per period, and resighted in subsequent periods. TOTAL1 shows the number of different individuals that were resighted at least once per “release” period. TOTAL2 shows the number of different individuals released and the number of different individuals observed per resighting period.

| **Birds wintering in France** | | | | |  | | | | |  | | | | |  | | | | |  | | | | |  | | | | |  | | | | |  | | | |  |
| --- | --- | --- | --- | --- | --- | --- | --- | --- | --- | --- | --- | --- | --- | --- | --- | --- | --- | --- | --- | --- | --- | --- | --- | --- | --- | --- | --- | --- | --- | --- | --- | --- | --- | --- | --- | --- | --- | --- | --- |
|  | | | | 2006 | | | | | | 2007 | | | | | 2008 | | | | | 2009 | | | | | 2010 | | | | | 2011 | | | | | 2012 | | | |  |
| Period | | | Released | w2 | | s1 | | s2 | w1 | | w2 | s1 | s2 | w1 | | w2 | s1 | s2 | w1 | | w2 | s1 | s2 | w1 | | w2 | s1 | s2 | w1 | | w2 | s1 | s2 | w1 | | w2 | s1 | s2 | TOTAL1 |
| 2005 | | w1 | 10 | 7 | | 6 | | 4 | 7 | | 9 | 3 | 6 | 7 | | 6 | 4 | 7 | 8 | | 6 | 4 | 3 | 5 | | 3 | 2 | 3 | 4 | | 4 | 3 | 3 | 5 | | 5 | 2 | 1 | 10 |
| 2006 | | w2 | 5 |  | | 2 | | 3 | 3 | | 3 | 2 | 0 | 2 | | 1 | 3 | 1 | 3 | | 1 | 3 | 2 | 0 | | 0 | 2 | 2 | 2 | | 0 | 1 | 1 | 2 | | 2 | 2 | 1 | 5 |
| 2006 | | w1 | 4 |  | |  | |  |  | | 2 | 1 | 3 | 3 | | 3 | 3 | 0 | 4 | | 2 | 1 | 1 | 3 | | 1 | 2 | 1 | 1 | | 2 | 2 | 2 | 1 | | 0 | 1 | 1 | 4 |
| 2007 | | w2 | 4 |  | |  | |  |  | |  | 1 | 2 | 3 | | 2 | 1 | 0 | 2 | | 1 | 1 | 1 | 1 | | 0 | 0 | 1 | 2 | | 2 | 1 | 1 | 2 | | 1 | 0 | 0 | 4 |
| 2007 | | w1 | 9 |  | |  | |  |  | |  |  |  |  | | 1 | 5 | 4 | 2 | | 0 | 5 | 6 | 4 | | 0 | 3 | 6 | 4 | | 2 | 5 | 5 | 5 | | 5 | 2 | 4 | 7 |
| 2008 | | w2 | 1 |  | |  | |  |  | |  |  |  |  | |  | 0 | 0 | 1 | | 1 | 0 | 0 | 1 | | 1 | 0 | 0 | 1 | | 1 | 0 | 0 | 0 | | 1 | 0 | 0 | 1 |
| 2008 | | w1 | 12 |  | |  | |  |  | |  |  |  |  | |  |  |  |  | | 9 | 5 | 7 | 11 | | 6 | 7 | 6 | 6 | | 7 | 7 | 5 | 8 | | 5 | 5 | 4 | 11 |
| 2009 | | w2 | 2 |  | |  | |  |  | |  |  |  |  | |  |  |  |  | |  | 1 | 1 | 0 | | 0 | 0 | 1 | 2 | | 2 | 1 | 1 | 1 | | 1 | 1 | 2 | 2 |
| 2009 | | w1 | 5 |  | |  | |  |  | |  |  |  |  | |  |  |  |  | |  |  |  |  | | 2 | 2 | 3 | 3 | | 3 | 4 | 2 | 4 | | 3 | 2 | 3 | 5 |
| 2010 | | w2 | 0 |  | |  | |  |  | |  |  |  |  | |  |  |  |  | |  |  |  |  | |  | 0 | 0 | 0 | | 0 | 0 | 0 | 0 | | 0 | 0 | 0 | 0 |
| 2010 | | w1 | 5 |  | |  | |  |  | |  |  |  |  | |  |  |  |  | |  |  |  |  | |  |  |  |  | | 2 | 2 | 2 | 3 | | 1 | 3 | 2 | 3 |
| 2011 | | w2 | 2 |  | |  | |  |  | |  |  |  |  | |  |  |  |  | |  |  |  |  | |  |  |  |  | |  | 2 | 1 | 0 | | 1 | 1 | 1 | 2 |
| 2011 | | w1 | 4 |  | |  | |  |  | |  |  |  |  | |  |  |  |  | |  |  |  |  | |  |  |  |  | |  |  |  |  | | 3 | 1 | 3 | 4 |
| 2012 | | w2 | 2 |  | |  | |  |  | |  |  |  |  | |  |  |  |  | |  |  |  |  | |  |  |  |  | |  |  |  |  | |  | 0 | 1 | 1 |
| TOTAL2 | | | 65 | 7 | | 8 | | 7 | 10 | | 14 | 7 | 11 | 15 | | 13 | 16 | 12 | 20 | | 20 | 20 | 21 | 25 | | 13 | 18 | 23 | 25 | | 25 | 28 | 23 | 31 | | 28 | 20 | 23 | 59 |
|  |  | |  |  | |  | |  |  | |  |  |  |  | |  |  |  |  | |  |  |  |  | |  |  |  |  | |  |  |  |  | |  |  |  |  |
| **Birds wintering in Iberia** | | | | |  | | | | |  | | | | |  | | | | |  | | | | |  | | | | |  | | | | |  | | | |  |
|  | | | | 2006 | | | | | | 2007 | | | | | 2008 | | | | | 2009 | | | | | 2010 | | | | | 2011 | | | | | 2012 | | | |  |
| Period | | | Released | w2 | | s1 | | s2 | w1 | | w2 | s1 | s2 | w1 | | w2 | s1 | s2 | w1 | | w2 | s1 | s2 | w1 | | w2 | s1 | s2 | w1 | | w2 | s1 | s2 | w1 | | w2 | s1 | s2 | TOTAL1 |
| 2005 | w1 | | 56 | 34 | | 29 | | 26 | 28 | | 16 | 25 | 31 | 24 | | 18 | 28 | 17 | 23 | | 13 | 24 | 29 | 15 | | 11 | 18 | 20 | 20 | | 11 | 23 | 21 | 20 | | 11 | 20 | 19 | 55 |
| 2006 | w2 | | 20 |  | | 10 | | 10 | 0 | | 3 | 8 | 8 | 4 | | 2 | 7 | 5 | 0 | | 5 | 6 | 8 | 3 | | 0 | 5 | 9 | 1 | | 0 | 6 | 4 | 3 | | 1 | 3 | 4 | 17 |
| 2006 | w1 | | 16 |  | |  | |  |  | | 2 | 6 | 9 | 5 | | 5 | 6 | 5 | 3 | | 3 | 8 | 7 | 5 | | 1 | 4 | 6 | 4 | | 2 | 4 | 7 | 4 | | 2 | 2 | 5 | 15 |
| 2007 | w2 | | 7 |  | |  | |  |  | |  | 2 | 1 | 0 | | 1 | 2 | 2 | 1 | | 0 | 2 | 1 | 0 | | 0 | 1 | 1 | 2 | | 1 | 2 | 2 | 1 | | 1 | 2 | 0 | 4 |
| 2007 | w1 | | 35 |  | |  | |  |  | |  |  |  |  | | 9 | 14 | 14 | 14 | | 8 | 14 | 14 | 7 | | 7 | 9 | 13 | 11 | | 5 | 10 | 16 | 10 | | 2 | 8 | 9 | 32 |
| 2008 | w2 | | 7 |  | |  | |  |  | |  |  |  |  | |  | 4 | 5 | 0 | | 0 | 6 | 5 | 1 | | 0 | 2 | 4 | 0 | | 1 | 4 | 4 | 1 | | 0 | 4 | 3 | 6 |
| 2008 | w1 | | 19 |  | |  | |  |  | |  |  |  |  | |  |  |  |  | | 7 | 8 | 10 | 7 | | 7 | 7 | 10 | 7 | | 6 | 9 | 9 | 6 | | 3 | 9 | 7 | 17 |
| 2009 | w2 | | 15 |  | |  | |  |  | |  |  |  |  | |  |  |  |  | |  | 7 | 12 | 3 | | 3 | 5 | 9 | 3 | | 0 | 5 | 8 | 2 | | 1 | 4 | 7 | 13 |
| 2009 | w1 | | 13 |  | |  | |  |  | |  |  |  |  | |  |  |  |  | |  |  |  |  | | 7 | 5 | 9 | 12 | | 6 | 8 | 9 | 12 | | 9 | 9 | 4 | 13 |
| 2010 | w2 | | 8 |  | |  | |  |  | |  |  |  |  | |  |  |  |  | |  |  |  |  | |  | 2 | 6 | 4 | | 4 | 5 | 3 | 1 | | 3 | 3 | 3 | 8 |
| 2010 | w1 | | 11 |  | |  | |  |  | |  |  |  |  | |  |  |  |  | |  |  |  |  | |  |  |  |  | | 3 | 10 | 6 | 5 | | 4 | 5 | 6 | 11 |
| 2011 | w2 | | 7 |  | |  | |  |  | |  |  |  |  | |  |  |  |  | |  |  |  |  | |  |  |  |  | |  | 2 | 2 | 2 | | 2 | 1 | 1 | 4 |
| 2011 | w1 | | 20 |  | |  | |  |  | |  |  |  |  | |  |  |  |  | |  |  |  |  | |  |  |  |  | |  |  |  |  | | 6 | 9 | 9 | 13 |
| 2012 | w2 | | 2 |  | |  | |  |  | |  |  |  |  | |  |  |  |  | |  |  |  |  | |  |  |  |  | |  |  |  |  | |  | 0 | 0 | 0 |
| TOTAL2 | | | 236 | 34 | | 39 | | 36 | 28 | | 21 | 41 | 49 | 33 | | 35 | 61 | 48 | 41 | | 36 | 75 | 86 | 41 | | 36 | 58 | 87 | 64 | | 39 | 88 | 91 | 67 | | 45 | 79 | 77 | 208 |
|  |  | |  |  | |  | |  |  | |  |  |  |  | |  |  |  |  | |  |  |  |  | |  |  |  |  | |  |  |  |  | |  |  |  |  |
| **Birds wintering in Mauritania** | | | | | | |  | | |  | | | | |  | | | | |  | | | | |  | | | | |  | | | | |  | | | |  |
|  | | | | 2006 | | | | | | 2007 | | | | | 2008 | | | | | 2009 | | | | | 2010 | | | | | 2011 | | | | | 2012 | | | |  |
| Period | | | Released | w2 | | s1 | | s2 | w1 | | w2 | s1 | s2 | w1 | | w2 | s1 | s2 | w1 | | w2 | s1 | s2 | w1 | | w2 | s1 | s2 | w1 | | w2 | s1 | s2 | w1 | | w2 | s1 | s2 | TOTAL1 |
| 2005 | w1 | | 10 | 1 | | 3 | | 4 | 3 | | 0 | 4 | 7 | 3 | | 0 | 3 | 4 | 0 | | 2 | 4 | 4 | 0 | | 0 | 2 | 3 | 1 | | 2 | 2 | 2 | 0 | | 0 | 0 | 1 | 9 |
| 2006 | w2 | | 15 |  | | 5 | | 2 | 3 | | 0 | 3 | 3 | 3 | | 0 | 2 | 1 | 1 | | 2 | 4 | 5 | 0 | | 0 | 1 | 2 | 1 | | 1 | 1 | 3 | 0 | | 1 | 4 | 2 | 11 |
| 2006 | w1 | | 28 |  | |  | |  |  | | 0 | 5 | 7 | 3 | | 5 | 5 | 2 | 5 | | 3 | 9 | 4 | 0 | | 0 | 3 | 7 | 1 | | 2 | 5 | 5 | 1 | | 2 | 3 | 2 | 19 |
| 2007 | w2 | | 7 |  | |  | |  |  | |  | 3 | 2 | 0 | | 0 | 4 | 2 | 0 | | 0 | 4 | 4 | 2 | | 0 | 1 | 0 | 0 | | 1 | 2 | 1 | 0 | | 1 | 2 | 1 | 4 |
| 2007 | w1 | | 48 |  | |  | |  |  | |  |  |  |  | | 12 | 8 | 11 | 13 | | 5 | 10 | 17 | 3 | | 2 | 12 | 19 | 7 | | 6 | 12 | 12 | 2 | | 6 | 8 | 8 | 36 |
| 2008 | w2 | | 10 |  | |  | |  |  | |  |  |  |  | |  | 1 | 1 | 1 | | 1 | 2 | 2 | 0 | | 0 | 3 | 3 | 3 | | 0 | 3 | 2 | 1 | | 2 | 2 | 2 | 5 |
| 2008 | w1 | | 22 |  | |  | |  |  | |  |  |  |  | |  |  |  |  | | 5 | 1 | 4 | 1 | | 0 | 7 | 11 | 2 | | 0 | 3 | 8 | 2 | | 4 | 6 | 5 | 17 |
| 2009 | w2 | | 20 |  | |  | |  |  | |  |  |  |  | |  |  |  |  | |  | 4 | 4 | 0 | | 0 | 3 | 5 | 1 | | 1 | 6 | 8 | 0 | | 2 | 2 | 4 | 11 |
| 2009 | w1 | | 9 |  | |  | |  |  | |  |  |  |  | |  |  |  |  | |  |  |  |  | | 0 | 1 | 3 | 1 | | 3 | 3 | 2 | 0 | | 1 | 1 | 3 | 6 |
| 2010 | w2 | | 0 |  | |  | |  |  | |  |  |  |  | |  |  |  |  | |  |  |  |  | |  | 0 | 0 | 0 | | 0 | 0 | 0 | 0 | | 0 | 0 | 0 | 0 |
| 2010 | w1 | | 16 |  | |  | |  |  | |  |  |  |  | |  |  |  |  | |  |  |  |  | |  |  |  |  | | 1 | 2 | 4 | 3 | | 4 | 2 | 2 | 12 |
| 2011 | w2 | | 20 |  | |  | |  |  | |  |  |  |  | |  |  |  |  | |  |  |  |  | |  |  |  |  | |  | 6 | 4 | 0 | | 1 | 3 | 5 | 8 |
| 2011 | w1 | | 4 |  | |  | |  |  | |  |  |  |  | |  |  |  |  | |  |  |  |  | |  |  |  |  | |  |  |  |  | | 2 | 0 | 0 | 2 |
| 2012 | w2 | | 28 |  | |  | |  |  | |  |  |  |  | |  |  |  |  | |  |  |  |  | |  |  |  |  | |  |  |  |  | |  | 2 | 7 | 8 |
| TOTAL2 | | | 237 | 1 | | 8 | | 6 | 6 | | 0 | 15 | 19 | 9 | | 17 | 23 | 21 | 20 | | 18 | 38 | 44 | 6 | | 2 | 33 | 53 | 17 | | 17 | 45 | 51 | 9 | | 26 | 35 | 42 | 148 |

**Table S2.** Data cloning results for Φ survival parameters of the full model (table 1, model 8). Estimates are on an annual basis and can be converted to 3-month (= 1/4 year) seasonal estimates by Φ^1/4^. 95% confidence intervals (CI) were estimated using the profile likelihood function.

|  | Wintering area | Original | | |  | Cloned (100x) | | |  | $\frac{\mathrm{SE}_{\mathrm{original}}}{\mathrm{SE}_{\mathrm{cloned}}}$ | $\frac{\mathrm{CI}_{\mathrm{original}}}{\mathrm{CI}_{\mathrm{cloned}}}$ |
| --- | --- | --- | --- | --- | --- | --- | --- | --- | --- | --- | --- |
| Season |  | mean | se | CI |  | mean | se | CI |  |  |  |
| Spring | France | 0.91 | 0.07 | 0.74-1.00 |  | 0.91 | 0.01 | 0.89-0.93 |  | 10.00 | 8.08 |
| Spring | Iberia | 0.84 | 0.04 | 0.75-0.92 |  | 0.84 | 0.00 | 0.83-0.85 |  | 10.00 | 8.69 |
| Spring | Mauritania | 0.43 | 0.04 | 0.36-0.51 |  | 0.43 | 0.00 | 0.42-0.44 |  | 10.00 | 8.60 |
| Summer | France | 1.00 | 0.00 | 0.88-1.00 |  | 1.00 | 0.00 | 1.00-1.00 |  | 6857.84 | 11323.62 |
| Summer | Iberia | 0.93 | 0.05 | 0.84-1.00 |  | 0.93 | 0.00 | 0.92-0.95 |  | 10.00 | 7.55 |
| Summer | Mauritania | 1.00 | 0.00 | 0.91-1.00 |  | 1.00 | 0.00 | 1.00-1.00 |  | 760.38 | 2155.70 |
| Autumn | France | 0.98 | 0.06 | 0.84-1.00 |  | 0.98 | 0.01 | 0.97-0.99 |  | 10.00 | 5.85 |
| Autumn | Iberia | 0.89 | 0.05 | 0.78-0.99 |  | 0.89 | 0.01 | 0.88-0.90 |  | 10.00 | 8.64 |
| Autumn | Mauritania | 1.00 | 0.00 | 0.91-1.00 |  | 1.00 | 0.00 | 1.00-1.00 |  | 306.70 | 1753.12 |
| Winter | France | 0.85 | 0.07 | 0.71-1.00 |  | 0.85 | 0.01 | 0.84-0.87 |  | 10.00 | 8.72 |
| Winter | Iberia | 1.00 | 0.00 | 0.91-1.00 |  | 1.00 | 0.00 | 1.00-1.00 |  | 17.43 | 108.43 |
| Winter | Mauritania | 1.00 | 0.00 | 0.91-1.00 |  | 1.00 | 0.00 | 1.00-1.00 |  | 665.08 | 2586.88 |

**Table S3.** Data cloning results for Φ parameters of the best-supported model (table 1, model 1). Estimates are on an annual basis and can be converted to 3-month (= 1/4 year) seasonal estimates by Φ^1/4^. 95% confidence intervals (CI) were estimated using the profile likelihood function.

|  | Wintering area | Original | | |  | Cloned (100x) | | |  | $\frac{\mathrm{SE}_{\mathrm{original}}}{\mathrm{SE}_{\mathrm{cloned}}}$ | $\frac{\mathrm{CI}_{\mathrm{original}}}{\mathrm{CI}_{\mathrm{cloned}}}$ |
| --- | --- | --- | --- | --- | --- | --- | --- | --- | --- | --- | --- |
| Season |  | mean | se | CI |  | mean | se | CI |  |  |  |
| Spring | France | 0.81 | 0.09 | 0.65-0.99 |  | 0.81 | 0.01 | 0.79-0.83 |  | 10.08 | 9.32 |
| Spring | Iberia | 0.79 | 0.05 | 0.70-0.90 |  | 0.79 | 0.00 | 0.78-0.80 |  | 10.06 | 9.49 |
| Spring | Mauritania | 0.46 | 0.05 | 0.37-0.57 |  | 0.46 | 0.00 | 0.45-0.47 |  | 10.02 | 9.87 |
| Summer |  | 0.97 | 0.03 | 0.89-1.00 |  | 0.97 | 0.00 | 0.96-0.98 |  | 10.00 | 7.31 |
| Autumn |  | 0.94 | 0.04 | 0.84-1.00 |  | 0.94 | 0.00 | 0.93-0.94 |  | 10.02 | 8.67 |
| Winter |  | 1.00 | 0.05 | 0.89-1.00 |  | 1.00 | 0.01 | 0.99-1.00 |  | 10.18 | 8.31 |

**
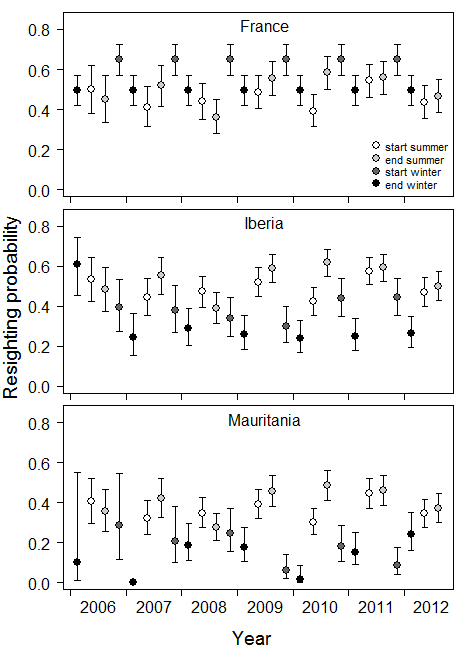
**

**Figure S1.** Resighting probabilities over time. Estimates are from the best-supported model (see table 1, model 1 of the manuscript). Error bars represent 95% confidence intervals, adjusted for overdispersion (ĉ=1.34).

## References

1. Leyrer J., Lok T., Brugge M., Dekinga A., Spaans B., Van Gils J.A., Sandercock B.K., Piersma T. 2012 Small-scale demographic structure suggests preemptive behavior in a flocking shorebird. *Behav. Ecol.* **23**, 1226-1233. (doi:10.1093/beheco/ars106)

2. Leyrer J., Lok T., Brugge M., Spaans B., Sandercock B.K., Piersma T. 2013 Mortality within the annual cycle: seasonal survival patterns in Afro-Siberian red knots *Calidris canutus canutus*. *J. Ornithol.* **154**, 933-943. (doi:10.1007/s10336-013-0959-y)

3. Lok T., Overdijk O., Tinbergen J.M., Piersma T. 2011 The paradox of spoonbill migration: most birds travel to where survival rates are lowest. *Anim. Behav.* **82**, 837-844. (doi:10.1016/j.anbehav.2011.07.019)

4. Lebreton J.D., Burnham K.P., Clobert J., Anderson D.R. 1992 Modeling survival and testing biological hypotheses using marked animals: a unified approach with case studies. *Ecol. Monogr.* **62**, 67-118. (doi:10.2307/2937171)

5. Hargrove J.W., Borland C.H. 1994 Pooled population parameter estimates from mark-recapture data. *Biometrics* **50**, 1129-1141.

6. O'Brien S., Robert B., Tiandry H. 2005 Consequences of violating the recapture duration assumption of mark-recapture models: a test using simulated and empirical data from an endangered tortoise population. *J. Appl. Ecol.* **42**, 1096-1104. (doi:10.1111/j.1365-2664.2005.01084.x)

7. Lok T., Overdijk O., Piersma T. 2013 Migration tendency delays distributional response to differential survival prospects along a flyway. *Am. Nat.* **181**, 520-531. (doi:10.1086/669679)

8. White G.C., Burnham K.P. 1999 Program MARK: survival estimation from populations of marked animals. *Bird Study* **46**, S120-S139.

9. Cooch E., White G.C. 2011 *Program MARK: a gentle introduction*. 11th ed. http://www.phidot.org/software/mark/docs/book/.

10. R Core Team. 2013 *R: A language and environment for statistical computing.* Vienna, Austria., R Foundation for Statistical Computing. http://www.R-project.org/.

11. Laake J.L. 2013 *RMark: An R Interface for Analysis of Capture-Recapture Data with MARK.* . 7600 Sand Point Way NE, Seattle WA 98115, Alaska Fish. Sci. Cent., NOAA, Natl. Mar. Fish. Serv.; 25 p.
